# The relationship of stress coping styles on substance use, depressive symptoms, and personality traits of nurses in higher education institution

**DOI:** 10.1002/npr2.12324

**Published:** 2023-03-05

**Authors:** Keita Tokumitsu, Norio Sugawara, Hiroaki Okayasu, Yasushi Kawamata, Masataka Shinozaki, Yoshiteru Sato, Aoi Sato, Yumiko Uchibori, Tomie Komatsu, Norio Yasui‐Furukori, Kazutaka Shimoda

**Affiliations:** ^1^ Department of Psychiatry, School of Medicine Dokkyo Medical University Mibu Japan; ^2^ Health Services Center for Students and Staff Dokkyo Medical University Mibu Japan; ^3^ Department of Nursing Dokkyo Medical University Hospital Mibu Japan

**Keywords:** brief COPE, depression, nurse, personality, substance use

## Abstract

**Aim:**

This study examined the collective characteristics of nurses and their stress coping style.

**Methods:**

We conducted a cluster analysis of the stress coping strategies of 841 nurses belonging to Dokkyo Medical University Hospital, as measured by the Brief COPE (Coping Orientation to Problems Experienced). We also conducted multivariate analyses of each cluster's sociodemographic characteristics, personality traits, depressive symptoms, work attitudes, sense of fairness, and turnover intention.

**Results:**

The results of cluster analysis using the standardized z scores of the Brief COPE demonstrated that the study participants were classified into three clusters. The “emotional‐response type” tended to favor the use of emotional support, venting, and self‐blame. The “reality‐escape type” tended to prefer alcohol and substance use, behavioral resignation, use of instrumental support, and lack of acceptance. The “problem‐solving type” tended to prefer planning, positive reframing, and acceptance and to dislike “alcohol and substance use” and behavioral disengagement. A multinomial logistic regression analysis revealed that compared to the problem‐solving type, the emotional‐response type had a lower job title, a higher “neuroticism” score on the TIPI‐J, and a higher K6 score. However, compared to the problem‐solving type, the reality‐escape type was younger, consumed more alcohol and substances, and had a higher K6 score.

**Conclusions:**

Stress coping styles were found to be associated with substance use, depressive symptoms, and personality traits among nurses in higher education institutions. Thus, the results suggest that nurses who choose maladaptive stress coping styles require mental support and early identification of depressive symptoms and alcohol problems.

## Abstract 2

We found that the nurses were most often of the “problem‐solving” type, who prefer rational responses to stressful situations and were considered to be adapting to the work environment as health care professionals. On the other hand, nurses of the emotional‐response type and reality‐escape types had higher K6 scores, an indicator of depression, than those of the problem‐solving type. In particular, the emotional‐response type had weak stress coping skills using interpersonal communication due to their personality tendencies, and the reality‐escape type was highlighted as being immature in age and at high risk for substance use disorders.

## Graphical abstract

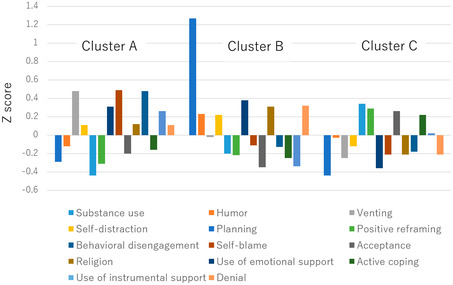

## 1 INTRODUCTION

The sophistication and specialization of medical technology, as well as the increasing efficiency of health care, requires nurses to demonstrate higher levels of job performance.^1^ With this in mind, it should also be noted that they are known to be at high risk for burnout,^2^ mood disorders,^3^ and substance use disorders^4^ due to irregular work schedules and high levels of physical and mental stress.^5^, ^6^ In an effort to improve occupational health conditions, stress checks are being conducted on hospital employees in Japan, including health care professionals, and occupational physicians are conducting interviews with employees who are under high psychological stress.^7^ In addition, mental health training and support are currently being provided by liaison nurses, psychologists and mental health managers to address this growing concern. Thus, stress management as an organizational initiative is becoming institutionalized, but when focusing on the self‐care of stress, the stress coping strategy preferences of nurses are not fully known, and there is a lack of research on their collective characteristics.

Humans cope with stress in a manner that is closely related to psychological defense mechanisms.^8^ Stress coping strategies have demonstrated importance in relation to psychosocial stress, depressive symptoms and suicidal ideation.^9^, ^10^ A more rational response to stress may be to reframe the problematic situation in a positive way, plan for it, and/or accept it. Conversely, more immature coping methods may involve the use of drugs, including alcohol, to escape stress or to engage in denial. In fact, health care workers, including nurses, have been reported to be at higher risk for substance use disorders than other professions.^4^ Avoidance and negative coping skills have also been linked to depression.^11^, ^12^ Alternatively, some staff may cope with stress by seeking access to emotional support or by actively expressing their feelings. Analyzing such coping preferences and the characteristics of their group attributes in relation to the intensity of psychological stress may help to build a more efficient support system.

The Brief COPE has been used as one of the rating scales in the study of coping styles.^13^ Fourteen items in total (10 items for positive coping and four items for negative coping) were used in the Brief COPE, and the Japanese version was validated. We observed the coping styles of nurses using the Brief COPE and conducted cluster analysis to examine whether there were differences in psychosocial backgrounds among the clusters. It has been reported that stress coping skills can be transformed through training.^14^ If we could clarify the differences in psychosocial backgrounds, including tendencies toward depression and personality traits, among the clusters aggregated by stress coping preferences, it would lead to the construction of more effective support systems for nurses, including education in using appropriate coping skills.

## 2 METHODS

### 2.1 Study design and subjects

This was a cross‐sectional observational study conducted from July to August 2020 using self‐administered questionnaires among 1063 nurses working at Dokkyo Medical University Hospital. Data from participants who answered at least age and gender were included in the analysis as valid responses. All participants were volunteers, there was no incentive to participate, and no exclusion criteria were established.

### 2.2 Study procedures

Nurses working at Dokkyo Medical University were asked to complete a self‐administered questionnaire. The questionnaire included the following items: characteristics of the nurses themselves (age, gender, marital status, smoking and drinking habits, presence or absence of children living with them, educational background, length of service, working hours, position, and night shift), personality traits using the Ten Item Personality Inventory (TIPI)‐J,^15^, ^16^ stress coping strategies using Brief COPE (Brief Coping Orientation to Problem Experienced),^13^ psychological stress using K6 (Kessler Psychological Distress Scale),^17^ attitude toward work using UWES (Utrecht Work Engagement Scale),^18^, ^19^ Moorman's organizational justice scale,^20^ and the Intention to Leave Scale.

The TIPI is a validated self‐rating questionnaire using the Big‐Five personality domains for evaluating personality traits.^15^ The TIPI evaluates five distinct personality domains: extraversion, agreeableness, conscientiousness, emotional stability, and openness to experience.^15^ The TIPI consists of 10 questions with a 7‐point Likert‐type scale. The scores for each five domains are scored on a 14‐point scale and represent how strongly each participant's personality fits into that domain.^15^ A higher score is defined as a stronger tendency toward them. In the TIPI personality domain, “emotional stability” was translated into Japanese as “*shinkeisho keiko* (neuroticism)” in the TIPI‐J. Note that “neuroticism” in the TIPI‐J is scored as the reversed‐score of “emotional stability” in the TIPI. In other words, the higher the “neuroticism” score in the TIPI‐J, the more emotionally unstable a person is considered.^21^, ^22^ The reliability and validity of the Japanese version of the TIPI (TIPI‐J) have been confirmed.^16^

The Brief COPE is a validated questionnaire consisting of 28 questions with a 4‐point Likert‐type scale that assesses 14 conceptually different coping mechanisms. The higher the score, the more frequently the coping skill is used.^13^ Coping skills include the following: positive mechanisms include active coping, planning, positive reframing, emotional support, religion, and instrumental support. On the other hand, negative mechanisms include self‐distraction, denial, venting, substance use, behavioral disengagement, and self‐blame.^13^ Humor and acceptance can be either positive or negative depending on their associated mechanisms.^13^ The reliability and validity of the Japanese version of the Brief COPE has been confirmed.^23^

The K6 is a self‐administered questionnaire that assesses participants' comprehensive psychological distress.^17^ The reliability and validity of the Japanese version of the K6 have been confirmed.^24^ The K6 consists of six questions, with higher K6 values indicating higher levels of psychological distress, and participants with a total K6 score greater than 13 were defined as having severe psychological distress.^25^

The Utrecht Work Engagement Scale was developed by Schaufeli et al.^18^ and assesses attitudes toward work. The present study assessed attitudes toward work using a shortened version of the Japanese translation of the Utrecht Work Engagement Scale (UWES‐J),^19^ which consists of three subfactors: vigor (3 items), dedication (3 items), and absorption (3 items). The UWES‐J was rated on a 7‐point Likert‐type scale, and higher scores indicated a stronger tendency toward these factors.^18^

Moorman's organizational justice scale is a self‐rating questionnaire that evaluates the perceived fairness of a supervisor's attitude and organizational management, and consists of two subscales (Procedural Justice and Interactional Justice).^20^ There were seven questions for the assessment of procedural justice and six questions for the assessment of interactional justice, each of which required responses on a 5‐point‐Likert type scale. Higher scores on each scale indicate that respondents believe their supervisors' attitudes and organizational management are fair. The Japanese version of Moorman's organizational justice scale, which has been confirmed to be reliable and valid,^26^ was used in this study.

The “intention to leave” of the study participants was measured by the original two items. The first question was “I intend to continue working at this hospital” and was rated using the four Likert options “strongly agree = 1”, “agree = 2”, “disagree = 3”, and “strongly disagree = 4”. The second question was “If I could change jobs under more favorable conditions, I would quit”, which was rated using four Likert options: “strongly agree = 4”, “agree = 3”, “disagree = 2”, and “strongly disagree = 1”. The total score was 8 points, and the higher the score, the higher the intention to retire was defined. In this study, we focused our analysis on the stress coping strategies of nurses.

### 2.3 Statistical analysis

All statistical analyses were performed with IBM SPSS Statistics version 28 for Microsoft Windows10 and EZR (Saitama Medical Center, Jichi Medical University, Saitama, Japan),^27^ which is a graphical user interface for R (The R Foundation for Statistical Computing, version 3.5.2). More precisely, EZR is a modified version of R Commander (version 2.5‐1) incorporating statistical functions that are frequently used in biostatistics.

All statistical tests were performed based on a two‐sided significance level of 0.05. First, descriptive statistics were performed for demographic and psychological characteristics. Then, Cronbach's alpha coefficient was used as a reliability indicator for the internal consistency of the Brief COPE scores in this study. The cases were cluster analyzed using the 14 items of the stress strategy as variables. Ward's method was used for clustering, Euclidean distance was used for scale sense, and a tree diagram was created. The standardization of values was transformed by Z scores for each case.

Then, based on the cluster classification, ANOVA was performed on the Z scores of the Brief COPE items in each cluster. We also performed a multiple comparison test for the Brief COPE subitems, applying Tukey's correction as a post hoc analysis of the ANOVA test.

In addition, differences in demographic and psychological characteristics on nominal variables of each cluster were identified using the chi‐square test (gender, smoking habit, drinking habit, number of children living together, educational background, length of service, working hours, job position, night shift work, proportion of K6 score ≥ 5, and K6 score ≥ 13). Additionally, differences in demographic and psychological characteristics on quantitative variables of each cluster were identified using Student's *t* test (Age, subitem of TIPI‐J, K6 score, subitem of UWES, subitem of Organizational justice, and Intention to leave).

After that, all factors were examined using multinomial logistic regression with forced imputation to ensure that potential associations were not overlooked. These factors included age, gender, marital status, smoking and drinking habits, presence or absence of children living with them, educational background, length of service, working hours, position, night shift availability, TIPI‐J, K6, UWES, and Moorman's organizational justice scale.

### 2.4 Ethics

This study was conducted in accordance with the Declaration of Helsinki and the Japanese Ethical Guidelines for Medical and Health Science Research Involving Human Subjects. Prior to the start of this study, the research protocol was reviewed and approved by the Institutional Review Board of the Ethics Committee of Dokkyo Medical University (Approval No. 29112). The research participants provided written agreement of cooperation, and the survey was conducted using a self‐administered questionnaire with no names. The completed forms were placed in a return envelope and deposited in a collection box, which was considered consent for the study. To access the data used in the study, our team obtained administrative privileges and licenses.

## 3 RESULTS

A total of 874 valid respondents completed the questionnaire including age and gender information. The response rate was 82.2% (874/1063). The sociodemographic characteristics of the valid respondents are shown in Table 1.

**TABLE 1:** Sociodemographic and characteristics of overall participants.

Factor	*n* (%)	Factor	Mean (SD)
Gender (%)		Age	34.20 (10.07)
Female	767 (87.8)	TIPI‐J	
Male	107 (12.2)	Openness to experience	7.48 (1.89)
Smoking habit (%)		Extraversion	8.59 (2.53)
Never smoked	668 (76.9)	Agreeableness	9.51 (1.81)
Current smoker	106 (12.2)	Conscientiousness	7.44 (2.06)
Past smoker	95 (10.9)	Neuroticism	8.57 (2.08)
Drinking habit (%)		Brief COPE	
≤2 times a week	686 (79.8)	Substance use	3.47 (1.61)
≥3 times a week	174 (20.2)	Humor	3.85 (1.27)
Number of Children living together (%)		Venting	5.20 (1.20)
None	536 (62.1)	Self‐distraction	5.30 (1.10)
One	125 (14.5)	Planning	5.53 (0.98)
More than two	202 (23.4)	Positive reframing	5.04 (1.07)
Educational background (%)		Behavioral disengagement	4.17 (0.99)
Nursing school	658 (75.5)	Self‐blame	5.11 (1.29)
Junior college	33 (3.8)	Acceptance	5.82 (0.89)
University	181 (20.8)	Religion	3.25 (1.23)
Length of service (%)		Use of emotional support	5.37 (1.25)
≥10 years	390 (44.8)	Active coping	5.46 (0.86)
<1 year	89 (10.2)	Use of instrumental support	5.63 (1.21)
≥1 year but <5 years	218 (25.1)	Denial	3.41 (1.16)
≥5 years but <10 years	173 (19.9)	K6 score	7.87 (5.72)
Working hours (%)		UWES	
40–49 h	430 (49.9)	Vigor (mean (SD))	5.39 (3.92)
<40 h	142 (16.5)	Dedication (mean (SD))	7.83 (3.82)
50–59 h	216 (25.1)	Absorption (mean (SD))	5.44 (3.94)
≥60 h	73 (8.5)	Organizational justice	
Job position (%)		Procedual justice	3.01 (0.75)
Rank and file member	767 (91.1)	Interactional justice	3.29 (0.89)
Chief	43 (5.1)	Intention to leave	5.74 (1.64)
Head Nurse or higher position	32 (3.8)		
Night shift work (%)			
No	133 (15.3)		
Yes	735 (84.7)		
K6 score ≥ 5 (%)			
No	275 (32.1)		
Yes	582 (67.9)		
K6 score ≥ 13 (%)			
No	688 (80.3)		
Yes	169 (19.7)		

Abbreviations: Brief COPE, Brief Coping Orientation to Problem Experienced; K6, the 6‐item Kessler Psychological Distress Scale; TIPI‐J, Japanese version of personality traits using the Ten Item Personality Inventory; UWES, Utrecht Work Engagement Scale.

Next, we conducted a cluster analysis of study participants, focusing on the Brief COPE. The Cronbach α coefficients for each factor were 0.63 (self‐distraction), 0.63 (active coping), 0.63 (denial), 0.65 (alcohol and substance use), 0.60 (use of emotional support), 0.61 (use of instrumental support), 0.64 (behavioral disengagement), 0.62 (venting), 0.62 (positive reframing), 0.63 (planning), 0.62 (humor), 0.64 (acceptance), 0.62 (religion), and 0.64 (self‐blame). In our study, the Cronbach's alpha coefficients for each Brief COPE item were above 0.6, which is considered to have good internal stability, reliability and acceptable consistency for an exploratory study.^28^

**FIGURE 1:**
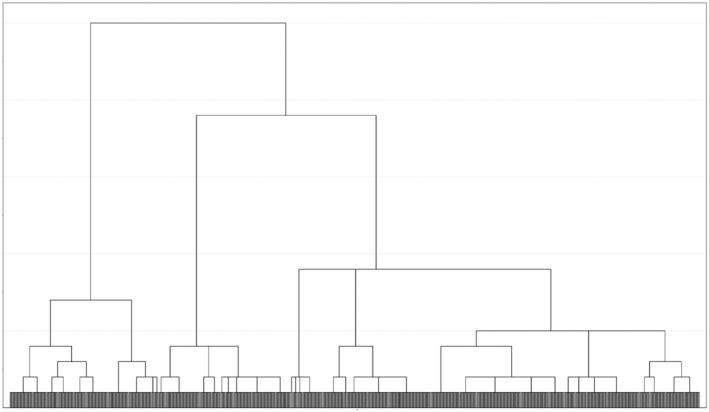
Dendrogram illustrated by hierarchical cluster analysis.

**FIGURE 2:**
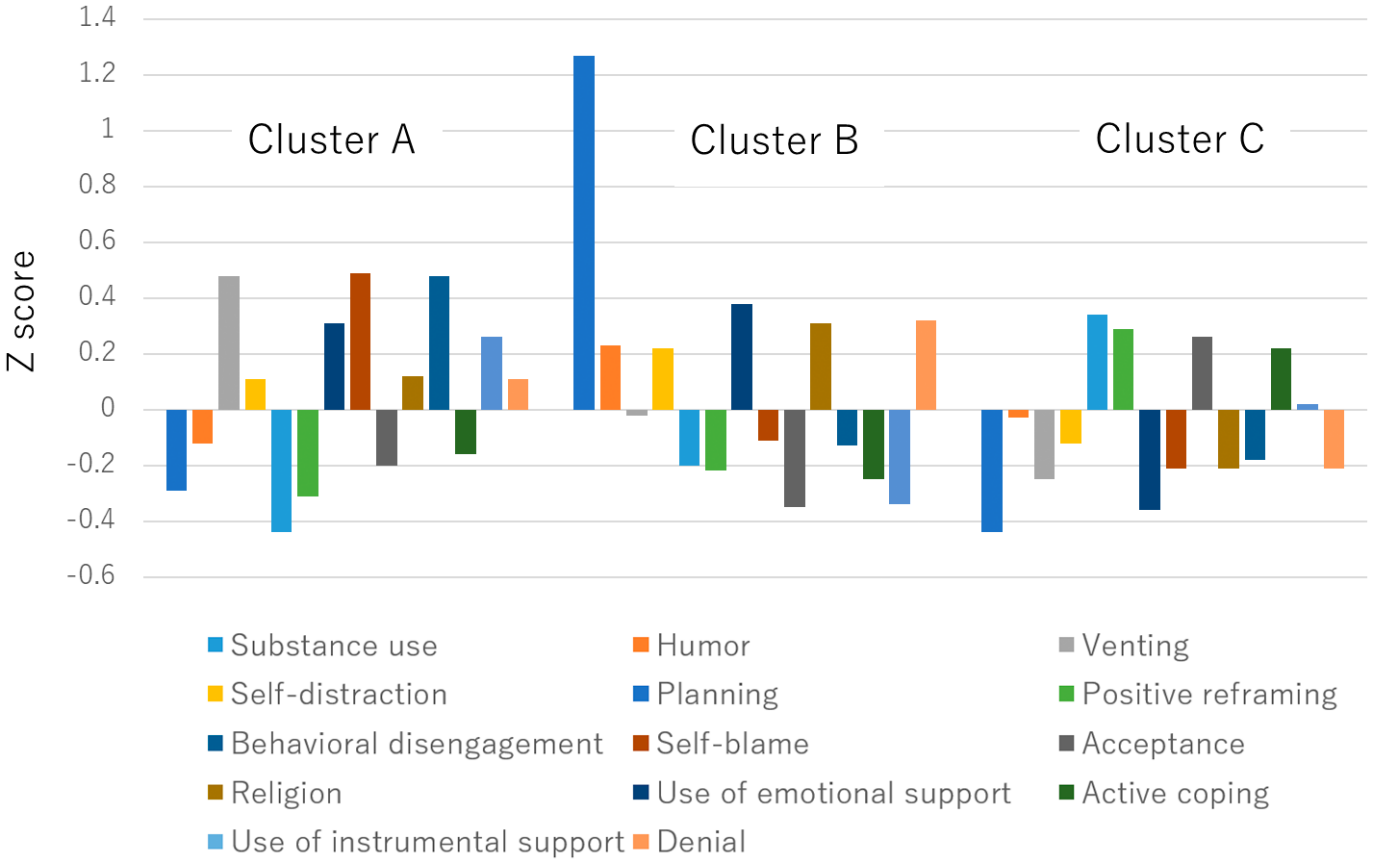
Distribution of the three clusters of the standardized variables derived from the brief COPE score.

The clustering process is illustrated in Figure 1. A total of 841 study participants completed the Brief Cope, and three possible clusters were identified based on the joining of branches. The structure of each cluster based on the standardized Z scores of the Brief COPE is also shown in Table 2 and Figure 2.

**TABLE 2:** Z scores of Brief COPE items in each cluster.

Factor	Cluster A	Cluster B	Cluster C	*p* value
	Emotional‐response	Reality‐escape	Problem‐solving	
*n*	227	192	422	
Substance use (mean (SD))	−0.29 (0.71)	1.27 (0.85)	−0.44 (0.62)	<0.001
Humor (mean (SD))	−0.12 (0.82)	0.23 (1.11)	−0.03 (1.02)	0.001
Venting (mean (SD))	0.48 (0.84)	−0.02 (1.07)	−0.25 (0.96)	<0.001
Self‐distraction (mean (SD))	0.11 (0.95)	0.22 (0.90)	−0.12 (1.03)	<0.001
Planning (mean (SD))	−0.44 (0.98)	−0.20 (1.12)	0.34 (0.80)	<0.001
Positive reframing (mean (SD))	−0.31 (1.00)	−0.22 (1.07)	0.29 (0.89)	<0.001
Behavioral disengagement (mean (SD))	0.31 (0.89)	0.38 (1.05)	−0.36 (0.90)	<0.001
Self‐blame (mean (SD))	0.49 (0.86)	−0.11 (0.99)	−0.21 (0.98)	<0.001
Acceptance (mean (SD))	−0.20 (1.01)	−0.35 (1.15)	0.26 (0.84)	<0.001
Religion (mean (SD))	0.12 (1.05)	0.31 (1.06)	−0.21 (0.89)	<0.001
Use of emotional support (mean (SD))	0.48 (0.92)	−0.13 (1.05)	−0.18 (0.94)	<0.001
Active coping (mean (SD))	−0.16 (0.95)	−0.25 (1.11)	0.22 (0.90)	<0.001
Use of instrumental support (mean (SD))	0.26 (0.97)	−0.34 (1.07)	0.02 (0.92)	<0.001
Denial (mean (SD))	0.11 (0.92)	0.32 (1.14)	−0.21 (0.92)	<0.001

Abbreviation: Brief COPE, Brief Coping Orientation to Problem Experienced.

According to these scores, Cluster A (*n* = 227) was higher than the other clusters in using emotional support, venting (expression of negative feelings), and self‐blaming. Cluster B (*n* = 192) was higher in alcohol and substance use, behavioral disengagement, and denial and lower in acceptance and use of instrumental support. Cluster C (*n* = 422) had higher levels of planning, positive reinterpretation, acceptance, and positive coping and lower levels of alcohol and substance use and behavioral disengagement. Based on these characteristics, the three clusters were named the emotional‐response type (Cluster A), reality‐escape type (Cluster B), and problem‐solving type (Cluster C). The sociodemographic characteristics of each cluster are shown in Table 3.

**TABLE 3:** Sociodemographic characteristics of each cluster of coping strategies.

Factor	Group	Emotional‐response	Reality‐escape	Problem‐solving	*p*‐value
*n*		227	192	422	
Gender (%)	Female	210 (92.5)	159 (82.8)	371 (87.9)	0.01
	Male	17 (7.5)	33 (17.2)	51 (12.1)	
Smoking habit (%)	Never smoked	188 (82.8)	125 (65.1)	332 (79.4)	<0.001
	Current smoker	20 (8.8)	36 (18.8)	44 (10.5)	
	Past smoker	19 (8.4)	31 (16.1)	42 (10.0)	
Drinking habit (%)	≤2 times a week	203 (91.0)	99 (52.7)	365 (87.5)	<0.001
	≥3 times a week	20 (9.0)	89 (47.3)	52 (12.5)	
Number of Children living together (%)	None	156 (69.0)	126 (67.0)	243 (58.3)	0.038
	One	23 (10.2)	23 (12.2)	71 (17.0)	
	More than two	47 (20.8)	39 (20.7)	103 (24.7)	
Educational background (%)	Nursing school	176 (77.9)	134 (69.8)	321 (76.2)	0.258
	Junior college	7 (3.1)	7 (3.6)	18 (4.3)	
	University	43 (19.0)	51 (26.6)	82 (19.5)	
Length of service (%)	≥10 years	93 (41.0)	77 (40.1)	201 (48.1)	0.183
	<1 year	29 (12.8)	15 (7.8)	42 (10.0)	
	≥1 year but <5 years	62 (27.3)	55 (28.6)	96 (23.0)	
	≥5 years but <10 years	43 (18.9)	45 (23.4)	79 (18.9)	
Working hours (%)	40–49 h	107 (47.6)	99 (52.7)	208 (50.1)	0.106
	<40 h	32 (14.2)	23 (12.2)	80 (19.3)	
	50–59 h	70 (31.1)	47 (25.0)	95 (22.9)	
	≥60 h	16 (7.1)	19 (10.1)	32 (7.7)	
Job position (%)	Rank and file member	206 (93.2)	172 (94.0)	362 (88.9)	0.016
	Chief	12 (5.4)	8 (4.4)	20 (4.9)	
	Head Nurse or higher position	3 (1.4)	3 (1.6)	25 (6.1)	
Night shift work (%)	No	27 (11.9)	16 (8.4)	83 (19.8)	<0.001
	Yes	199 (88.1)	174 (91.6)	336 (80.2)	
K6 score ≥ 5 (%)	No	46 (20.4)	44 (23.3)	177 (42.5)	<0.001
	Yes	179 (79.6)	145 (76.7)	239 (57.5)	
K6 score ≥ 13 (%)	No	158 (70.2)	141 (74.6)	367 (88.2)	<0.001
	Yes	67 (29.8)	48 (25.4)	49 (11.8)	
Age (mean (SD))		32.63 (9.17)	32.56 (8.84)	35.32 (10.65)	<0.001
TIPI‐J	Openness to Experience (mean (SD))	7.03 (1.92)	7.41 (1.81)	7.73 (1.88)	<0.001
	Extraversion (mean (SD))	7.91 (2.48)	8.54 (2.56)	8.91 (2.44)	<0.001
	Agreeableness (mean (SD))	9.53 (1.84)	9.12 (1.86)	9.74 (1.69)	<0.001
	Conscientiousness (mean (SD))	6.94 (2.04)	7.06 (1.99)	7.91 (1.98)	<0.001
	Neuroticism (mean (SD))	9.50 (2.15)	8.69 (2.05)	8.06 (1.88)	<0.001
K6 score (mean (SD))		9.72 (5.71)	9.10 (5.89)	6.32 (5.19)	<0.001
UWES	Vigor (mean (SD))	4.86 (3.61)	4.43 (3.79)	6.03 (3.95)	<0.001
	Dedication (mean (SD))	7.37 (3.68)	6.77 (3.91)	8.53 (3.63)	<0.001
	Absorption (mean (SD))	5.05 (3.84)	4.60 (3.66)	5.97 (3.91)	<0.001
Organizational justice	Procedual justice (mean (SD))	3.01 (0.72)	2.94 (0.79)	3.05 (0.73)	0.248
	Interactional justice (mean (SD))	3.32 (0.84)	3.09 (0.97)	3.37 (0.85)	0.001
Intention to leave (mean (SD))		5.98 (1.61)	6.03 (1.61)	5.50 (1.63)	<0.001

Abbreviation: K6, the 6‐item Kessler Psychological Distress Scale.

We summarized the results of multiple comparison tests applying Tukey's correction as a post hoc analysis of the ANOVA test for the Z score of each Brief COPE item in each cluster in Table 4. For example, the mean Z score for the “use of instrumental support” is 0.26 for cluster A, −0.34 for cluster B, and 0.02 for cluster C (Table 2). Considering the results of the multiple comparison test, we found that the mean Z score for the “use of instrumental support” in cluster A is statistically significantly greater than in clusters B and C (Similarly, we found a statistically significant difference between clusters B and C; Table 4).

**TABLE 4:** Multiple comparison tests applying Tukey's correction for the Z score of each Brief COPE item in each cluster.

Dependent variable	(I) cluster	(J) cluster	Mean difference (I‐J)	Std. error	*p* value	95% confidence interval for mean difference	
						Lower bound	Upper bound
Self‐distraction	Cluster A	Cluster B	−0.108	0.096	0.501	−0.334	0.118
		Cluster C	0.231	0.081	0.012	0.042	0.421
	Cluster B	Cluster A	0.108	0.096	0.501	−0.118	0.334
		Cluster C	0.339	0.085	<0.001	0.139	0.540
	Cluster C	Cluster A	−0.231	0.081	0.012	−0.421	−0.042
		Cluster B	−0.339	0.085	<0.001	−0.540	−0.139
Active coping	Cluster A	Cluster B	0.088	0.094	0.621	−0.134	0.309
		Cluster C	−0.385	0.079	<0.001	−0.571	−0.199
	Cluster B	Cluster A	−0.088	0.094	0.621	−0.309	0.134
		Cluster C	−0.473	0.084	<0.001	−0.669	−0.276
	Cluster C	Cluster A	0.385	0.079	<0.001	0.199	0.571
		Cluster B	0.473	0.084	<0.001	0.276	0.669
Denial	Cluster A	Cluster B	−0.211	0.096	0.071	−0.435	0.014
		Cluster C	0.317	0.080	<0.001	0.129	0.506
	Cluster B	Cluster A	0.211	0.096	0.071	−0.014	0.435
		Cluster C	0.528	0.085	<0.001	0.329	0.727
	Cluster C	Cluster A	−0.317	0.080	<0.001	−0.506	−0.129
		Cluster B	−0.528	0.085	<0.001	−0.727	−0.329
Substance use	Cluster A	Cluster B	−1.557	0.069	<0.001	−1.718	−1.395
		Cluster C	0.154	0.058	0.021	0.019	0.290
	Cluster B	Cluster A	1.557	0.069	<0.001	1.395	1.718
		Cluster C	1.711	0.061	<0.001	1.568	1.855
	Cluster C	Cluster A	−0.154	0.058	0.021	−0.290	−0.019
		Cluster B	−1.711	0.061	<0.001	−1.855	−1.568
Use of emotional support	Cluster A	Cluster B	0.619	0.094	<0.001	0.398	0.839
		Cluster C	0.666	0.079	<0.001	0.481	0.851
	Cluster B	Cluster A	−0.619	0.094	<0.001	−0.839	−0.398
		Cluster C	0.047	0.083	0.839	−0.149	0.243
	Cluster C	Cluster A	−0.666	0.079	<0.001	−0.851	−0.481
		Cluster B	−0.047	0.083	0.839	−0.243	0.149
Use of instrumental support	Cluster A	Cluster B	0.605	0.095	<0.001	0.381	0.829
		Cluster C	0.240	0.080	0.008	0.052	0.428
	Cluster B	Cluster A	−0.605	0.095	<0.001	−0.829	−0.381
		Cluster C	−0.365	0.085	<0.001	−0.564	−0.167
	Cluster C	Cluster A	−0.240	0.080	0.008	−0.428	−0.052
		Cluster B	0.365	0.085	<0.001	0.167	0.564
Behavioral disengagement	Cluster A	Cluster B	−0.077	0.091	0.680	−0.291	0.138
		Cluster C	0.663	0.077	<0.001	0.483	0.843
	Cluster B	Cluster A	0.077	0.091	0.680	−0.138	0.291
		Cluster C	0.740	0.081	<0.001	0.549	0.930
	Cluster C	Cluster A	−0.663	0.077	<0.001	−0.843	−0.483
		Cluster B	−0.740	0.081	<0.001	−0.930	−0.549
Venting	Cluster A	Cluster B	0.498	0.094	<0.001	0.278	0.718
		Cluster C	0.727	0.079	<0.001	0.543	0.912
	Cluster B	Cluster A	−0.498	0.094	<0.001	−0.718	−0.278
		Cluster C	0.229	0.083	0.016	0.034	0.425
	Cluster C	Cluster A	−0.727	0.079	<0.001	−0.912	−0.543
		Cluster B	−0.229	0.083	0.016	−0.425	−0.034
Positive reflaming	Cluster A	Cluster B	−0.086	0.094	0.630	−0.308	0.135
		Cluster C	−0.599	0.079	<0.001	−0.785	−0.413
	Cluster B	Cluster A	0.086	0.094	0.630	−0.135	0.308
		Cluster C	−0.513	0.084	<0.001	−0.709	−0.316
	Cluster C	Cluster A	0.599	0.079	<0.001	0.413	0.785
		Cluster B	0.513	0.084	<0.001	0.316	0.709
Planning	Cluster A	Cluster B	−0.233	0.091	0.029	−0.446	−0.019
		Cluster C	−0.778	0.076	<0.001	−0.958	−0.599
	Cluster B	Cluster A	0.233	0.091	0.029	0.019	0.446
		Cluster C	−0.546	0.081	<0.001	−0.735	−0.356
	Cluster C	Cluster A	0.778	0.076	<0.001	0.599	0.958
		Cluster B	0.546	0.081	<0.001	0.356	0.735
Humor	Cluster A	Cluster B	−0.346	0.097	0.001	−0.575	−0.118
		Cluster C	−0.085	0.082	0.554	−0.276	0.107
	Cluster B	Cluster A	0.346	0.097	0.001	0.118	0.575
		Cluster C	0.262	0.086	0.007	0.059	0.465
	Cluster C	Cluster A	0.085	0.082	0.554	−0.107	0.276
		Cluster B	−0.262	0.086	0.007	−0.465	−0.059
Acceptance	Cluster A	Cluster B	0.155	0.094	0.230	−0.067	0.376
		Cluster C	−0.456	0.079	<0.001	−0.642	−0.270
	Cluster B	Cluster A	−0.155	0.094	0.230	−0.376	0.067
		Cluster C	−0.610	0.084	<0.001	−0.807	−0.414
	Cluster C	Cluster A	0.456	0.079	<0.001	0.270	0.642
		Cluster B	0.610	0.084	<0.001	0.414	0.807
Religion	Cluster A	Cluster B	−0.183	0.096	0.136	−0.407	0.042
		Cluster C	0.331	0.080	<0.001	0.142	0.519
	Cluster B	Cluster A	0.183	0.096	0.136	−0.042	0.407
		Cluster C	0.514	0.085	<0.001	0.314	0.713
	Cluster C	Cluster A	−0.331	0.080	<0.001	−0.519	−0.142
		Cluster B	−0.514	0.085	<0.001	−0.713	−0.314
Self‐blame	Cluster A	Cluster B	0.605	0.093	<0.001	0.386	0.825
		Cluster C	0.703	0.078	<0.001	0.519	0.888
	Cluster B	Cluster A	−0.605	0.093	<0.001	−0.825	−0.386
		Cluster C	0.098	0.083	0.465	−0.097	0.293
	Cluster C	Cluster A	−0.703	0.078	<0.001	−0.888	−0.519
		Cluster B	−0.098	0.083	0.465	−0.293	0.097

For each cluster, the results of multinomial logistic regression analysis with the problem‐solving type as the reference are shown in Table 5. The results of multinomial logistic regression analysis demonstrated that, compared to the problem‐solving type, the emotional‐response type had a higher score of neuroticism on the TIPI‐J (OR = 1.214, *p* = 0.001) and a higher K6 score (OR = 1.101, *p* < 0.001). In addition, the emotional‐response type had a significantly higher percentage of rank‐and‐file members than the problem‐solving type when the head nurse or higher position was set as a reference (OR = 5.986, *p* = 0.039). Compared to the problem‐solving type, the reality‐escape type was younger (OR = 0.947, *p* = 0.029), had a higher K6 score (OR = 1.078, *p* = 0.002), and had a drinking habit (OR = 8.920, *p* < 0.001).

**TABLE 5:** Multinomial logistic regression analysis for each factor across clusters of coping strategies.

Factor	Emotional‐response type					Reality‐escape type				
	B	Std. error	Wald	Odds ratio(95% CI)	*p*‐value	B	Std. error	Wald	Odds ratio(95% CI)	*p*‐value
Intercept	−2.944	1.956	2.265	(−)	0.132	2.092	2.189	0.913	(−)	0.339
Age	−0.020	0.020	0.982	0.980 (0.943–1.020)	0.322	−0.054	0.025	4.747	0.947 (0.902–0.995)	0.029
TIPI‐J										
Extraversion	−0.029	0.045	0.411	0.972 (0.890–1.061)	0.521	−0.046	0.051	0.844	0.955 (0.864–1.054)	0.358
Agreeableness	−0.031	0.061	0.255	0.969 (0.860–1.093)	0.613	−0.126	0.070	3.274	0.882 (0.769–1.011)	0.070
Conscientiousness	−0.109	0.056	3.778	0.897 (0.804–1.001)	0.052	−0.081	0.064	1.590	0.923 (0.814–1.046)	0.207
Neuroticism	0.194	0.059	10.713	1.214 (1.081–1.363)	0.001	0.013	0.067	0.038	1.013 (0.889–1.155)	0.845
Openness to Experience	−0.111	0.058	3.720	0.895 (0.799–1.002)	0.054	−0.100	0.067	2.245	0.905 (0.794–1.031)	0.134
K6 score	0.096	0.022	19.274	1.101 (1.055–1.150)	0.000	0.075	0.024	9.561	1.078 (1.028–1.130)	0.002
UWES										
Vigor	0.075	0.049	2.416	1.078 (0.981–1.186)	0.120	0.018	0.055	0.107	1.018 (0.914–1.134)	0.743
Dedication	−0.048	0.049	0.976	0.953 (0.866–1.049)	0.323	−0.075	0.057	1.706	0.928 (0.830–1.038)	0.192
Absorption	−0.037	0.046	0.672	0.963 (0.881–1.053)	0.412	−0.002	0.052	0.001	0.998 (0.901–1.106)	0.971
Organizational justice										
Procedual justice	0.024	0.165	0.021	1.024 (0.741–1.416)	0.885	0.213	0.188	1.281	1.237 (0.856–1.790)	0.258
Interactional justice	0.079	0.143	0.301	1.082 (0.817–1.433)	0.583	−0.020	0.154	0.017	0.980 (0.725–1.326)	0.898
Intention to leave	0.054	0.075	0.511	1.055 (0.911–1.223)	0.475	0.133	0.087	2.333	1.142 (0.963–1.355)	0.127
Gender										
Female	0.645	0.356	3.280	1.907 (0.948–3.833)	0.070	0.141	0.339	0.172	1.151 (0.592–2.239)	0.678
Male	Reference within this factor			(−)		Reference within this factor			(−)	
Number of Children living together										
None	−0.033	0.302	0.012	0.968 (0.535–1.750)	0.914	0.084	0.352	0.057	1.087 (0.546–2.166)	0.812
One	−0.442	0.371	1.421	0.643 (0.311–1.329)	0.233	0.062	0.405	0.024	1.064 (0.481–2.354)	0.878
More than two	Reference within this factor			(−)		Reference within this factor			(−)	
Educational background										
Nursing school	0.192	0.262	0.534	1.211 (0.725–2.024)	0.465	−0.387	0.278	1.930	0.679 (0.394–1.172)	0.165
Junior college	−0.132	0.569	0.053	0.877 (0.287–2.674)	0.817	−0.148	0.614	0.058	0.862 (0.259–2.873)	0.809
University	Reference within this factor			(−)		Reference within this factor			(−)	
Length of service										
<1 year	−0.734	0.501	2.151	0.480 (0.180–1.280)	0.142	−0.656	0.577	1.292	0.519 (0.167–1.608)	0.256
≥1 year but <5 years	−0.371	0.395	0.886	0.690 (0.318–1.495)	0.347	−0.457	0.465	0.964	0.633 (0.254–1.576)	0.326
≥5 years but <10 years	−0.407	0.356	1.309	0.665 (0.331–1.337)	0.253	−0.196	0.397	0.244	0.822 (0.378–1.789)	0.621
≥10 years	Reference within this factor			(−)		Reference within this factor			(−)	
Working hours										
<40 h	0.159	0.484	0.108	1.173 (0.454–3.027)	0.742	−0.111	0.530	0.044	0.895 (0.317–2.529)	0.834
40–49 h	0.304	0.410	0.548	1.355 (0.606–3.027)	0.459	0.045	0.433	0.011	1.046 (0.448–2.442)	0.918
50–59 h	0.471	0.427	1.214	1.601 (0.693–3.701)	0.271	−0.124	0.467	0.071	0.883 (0.354–2.205)	0.790
≥ 60 hours	Reference within this factor			(−)		Reference within this factor			(−)	
Job position										
Rank and file member	1.789	0.865	4.278	5.986 (1.098–32.630)	0.039	0.018	0.789	0.001	1.018 (0.217–4.775)	0.982
Chief	1.652	0.925	3.189	5.220 (0.851–32.012)	0.074	0.214	0.880	0.059	1.239 (0.221–6.956)	0.808
Head Nurse or higher position	Reference within this factor			(−)		Reference within this factor			(−)	
Smoking habit										
Current smoker	−0.366	0.387	0.894	0.694 (0.325–1.481)	0.344	0.302	0.366	0.680	1.352 (0.660–2.769)	0.410
Past smoker	−0.048	0.358	0.018	0.953 (0.472–1.924)	0.894	0.578	0.364	2.520	1.782 (0.873–3.636)	0.112
Never smoked	Reference within this factor			(−)		Reference within this factor			(−)	
Drinking habit										
≥3 times a week	−0.112	0.341	0.109	0.894 (0.458–1.744)	0.742	2.188	0.281	60.493	8.920 (5.139–15.482)	0.000
≤2 times a week	Reference within this factor			(−)		Reference within this factor			(−)	
Night shift work										
No	−0.603	0.358	2.839	0.547 (0.271–1.103)	0.092	−0.537	0.406	1.744	0.585 (0.264–1.297)	0.187
Yes	Reference within this factor			(−)		Reference within this factor			(−)	

*Note*: Reference group: Problem‐solving type.

Abbreviations: Brief COPE, Brief Coping Orientation to Problem Experienced; K6, the 6‐item Kessler Psychological Distress Scale; TIPI‐J, Japanese version of personality traits using the Ten Item Personality Inventory; UWES, Utrecht Work Engagement Scale.

## 4 DISCUSSION

The current study, which focused on the stress coping styles of nurses, found that nurses could be classified into three clusters. This was consistent with previous research that has shown that coping categories can be divided into three types: problem‐solving, emotional distraction, and avoidant strategies.^29^ In our study, more than half of the nurses belonged to the problem‐solving type, which was considered to have a rational attitude toward resolving stressful situations through positive coping and adapting to the work environment. On the other hand, compared to the problem‐solving type, the emotional‐response and reality‐escape types tended to use negative coping, which was found to be associated with depressive symptoms. This result appears to be consistent with previous research showing that maladaptive coping is a predictor of depression, anxiety and stress^30^ and that problem‐solving strategies are associated with a lower risk of depression.^31^

In a previous study of nurses, the prevalence of depressive symptoms was reported to be higher in nurses who held lower positions and drank more.^32^ Since psychological stress has been reported to be associated with the occurrence of medical accidents among nurses,^33^ a mental support system focusing on the stress coping strategies of nurses was also considered necessary for medical safety.

In particular, it was highlighted that the study participants belonging to the reality‐escape type were significantly more likely to have drinking habits than those belonging to the problem‐solving type (OR = 8.920, *p* < 0.001). Previous studies have also shown that people who choose avoidant stress coping tend to prefer drinking.^34^ It has been suggested that nurses who are unable to implement positive stress management strategies in high‐stress situations may be self‐medicating their psychological distress by drinking.^35^ In addition, substance use disorders and major depression are closely related.^36^ However, coping skills training can be effective in correcting drinking behavior.^37^, ^38^ For this reason, it was thought that more active psychological support was needed for nurses who chose the escape from reality coping style.

We also found that the reality‐escape type was associated with lower age. Due to inexperience, they may not be able to acquire a rational coping style and may suffer from adjustment disorders in the workplace. In addition, stress management training programs reduce occupational stress and improve coping strategies in nurses.^14^ For this reason, it seemed advisable to provide opportunities for stress coping learning, especially in the training of new employees.

It was also found that when a head nurse or higher position was used as the reference for a job position, the study participants belonging to the emotional‐response type had a significantly higher percentage of rank‐and‐file members compared to the problem‐solving type (OR = 5.986, *p* = 0.039). A previous study reported that the coping style of the emotionally reactive type has a strong tendency toward self‐criticism and weak planning, which may lead to burnout and resignation.^39^ This suggests that nurses may not be able to perform positive and proper stress coping and may leave their jobs early.

In addition, the emotional‐response type was found to have higher scores on the TIPI‐J on “Neuroticism” and higher scores on the Brief COPE on “Venting” and “Self‐blaming”. These results suggest that the emotional‐response type perceives themselves as emotionally unstable, may have low stress tolerance and experience difficulties in career development given their stress coping strategies. On the other hand, the inability to appropriately cope with stress in the workplace may cause depression and self‐blaming. These findings suggest the need for organization‐wide stress management that takes into account stress coping styles and personality tendencies.

There are several limitations to our study. This study is an observational study of nurses at Dokkyo Medical University Hospital. Because the participant population belongs to a single institution, population bias may have occurred, and the study results cannot be generalized to all nurses. Additionally, this study was a cross‐sectional observational study. Since it is not a longitudinal study, the causal relationship between independent variables and dependent variables is unknown. Therefore, a longitudinal study of other institutions regarding the coping styles of nurses was deemed necessary.

Regarding the independent variable, the Resignation Rating Scale is a unique scale that we devised. Since the relationship between actual resignation and the resignation rating scale has not been fully elucidated, caution is required in interpreting the results.

Our findings suggest that improving stress coping skills based on the stratification of coping strategies is effective in the management of nurses in higher health care organizations, but additional intervention studies should be conducted with a longitudinal design to build more robust evidence.

## 5 CONCLUSIONS

Stress coping styles were found to be associated with substance use, depressive symptoms, and personality traits among nurses in higher education institutions. Thus, the results suggest that nurses who choose maladaptive stress coping styles require mental support and early identification of depressive symptoms and alcohol problems.

## AUTHOR CONTRIBUTIONS

KT performed statistical analyses and wrote the first draft of the manuscript. NS and NYF contributed to the conception and design of the work. YU and TK were involved in the data acquisition. NS, HO, YK, MS, YS, AS, YU, TK, NYF and KS contributed to the critical review of the manuscript. All authors approved the final version of the manuscript.

## FUNDING INFORMATION

This study was supported by the Japan Agency for Medical Research and Development (AMED) under grant numbers JP19dk0307083 and JP20dk0307081, Health and Labor Sciences Research Grants (19GC1201) and Grants‐in‐Aid for Scientific Research (KAKENHI: 20K07134, 21K07486) from the Japan Society for the Promotion of Research JSPS. The funders had no role in the study design, data collection and analysis, decision to publish, or preparation of the manuscript.

## CONFLICT OF INTEREST STATEMENT

Norio Yasui‐Furukori has been a speaker for Dainippon‐Sumitomo Pharmaceutical, Mochida Pharmaceutical, and MSD. Kazutaka Shimoda has received research support from Meiji Seika Pharma Co., Pfizer Inc., Dainippon Sumitomo Pharma Co., Ltd., Daiichi Sankyo Co., Otsuka Pharmaceutical Co., Ltd., Astellas Pharma Inc., Novartis Pharma K.K., Eisai Co., Ltd., Takeda Pharmaceutical Co., Ltd. and honoraria from Mitsubishi Tanabe Pharma Corporation, Meiji Seika Pharma Co., Ltd., Dainippon Sumitomo Pharma Co., Ltd., Takeda Pharmaceutical Co., Shionogi & Co., Ltd., Daiichi Sankyo Co., Pfizer Inc. and Eisai Co., Ltd. These companies had no role in the study design, the data collection and analysis, the decision to publish, or the preparation of the manuscript. The remaining authors declare no conflict of interest.

## ETHICAL APPROVAL

This study was conducted in accordance with the Declaration of Helsinki and the Japanese Ethical Guidelines for Medical and Health Science Research Involving Human Subjects. Prior to the start of this study, the research protocol was reviewed and approved by the Institutional Review Board of the Ethics Committee of Dokkyo Medical University (Approval No. 29112).

Patient Consent Statement: The research subjects were requested to cooperate in writing using a cooperation request form, and the survey was conducted using a self‐administered questionnaire with no names. The completed forms were placed in a return envelope and deposited in a collection box, which was considered consent for the study. To access the data used in the study, our team obtained administrative privileges and licenses.

Registry and the Registration No. of the Study/Trial: N/A.

Animal Studies: N/A.

## Data Availability

The institutional review board of the ethics committee of Dokkyo Medical University set restrictions on data sharing because the data contain potentially identifying or sensitive participant information. The database was approved by the research ethics committee. Please contact the corresponding author when requesting data.
